# Anti-inflammatory effects of Jingshu Keli capsule and its components on human synoviocyte MH7A cells

**DOI:** 10.1186/s42836-020-00026-8

**Published:** 2020-03-09

**Authors:** Xiangbo Meng, Wenxiang Cheng, Shan Zhong, Peng Zhang, Ling Qin, Xinluan Wang

**Affiliations:** 1 Translational Medicine R&D Center, Shenzhen Institutes of Advanced Technology, Chinese Academy of Sciences, Shenzhen, 518055 China; 2 Musculoskeletal Research Laboratory, Department of Orthopaedics and Traumatology, The Chinese University of Hong Kong, Hong Kong, SAR China

**Keywords:** *Jingshu Keli*, Single-herb capsules, Single-herb-deleted capsules, Anti-inflammatory

## Abstract

**Background:**

*Jingshu Keli* (JSKL), a traditional Chinese medicine (TCM) formula consisting of multiple active compounds, has been officially approved by National Medical Products Administration (NMPA) for treatment of cervical radiculopathy. It relieves pain, according to TCM theory, by activating blood circulation to dissipate blood stasis. The pain mainly stems from neurogenic inflammation caused by mechanical compression of the cervical nerve root. In addition, inflammation mediators also cause the development of other joint diseases, such as osteoarthritis (OA). The purpose of this paper was to evaluate the anti-inflammatory effects of JSKL and identify the biologically active herbs and compounds *in vitro*.

**Methods:**

Enzyme-linked immunosorbent assay (Elisa) was used to determine the expression of pro-inflammatory cytokines, tumor necrosis factor-alpha (TNF-α), interleukin 6 (IL-6) and interleukin 8 (IL-8), in the culture medium of human MH7A cells stimulated by lipopolysaccharides (LPS).

**Results:**

JSKL and three single-herb capsules, *Cinnamomum cassia* Presl (*C.C.*), *Angelica Sinensis* (Oliv.) Diels (*A.S*.) and *Carthamus tinctorius* L. (*C.T.*), significantly inhibited the secretion of TNF-α. If one of these three herbal components was removed, suppressing effect of the single-herb-deleted JSKL on TNF-α was abolished. Cinnamaldehyde (CIN) from *C.C.* was the most potent ingredient that inhibited the expression of IL-6 and IL-8 in the culture medium of both LPS-stimulated MH7A cells and primary synovial cells.

**Conclusions:**

JSKL was found to possess anti-inflammatory effect *in vitro*; *C.C.*, *A.S.* and *C.T.* were the principal and essential herbal components responsible for such activity; CIN from *C.C.* is one the most potent single compound among indicator components of JSKL recorded in 2015 Chinese pharmacopoeia. This study provided scientific evidence for the clinical application of JSKL as an agent for targeted treatment of cervical radiculopathy. Furthermore, CIN has potential to be used for the treatment of some inflammation-related orthopedic diseases, such as rheumatic arthritis and osteoarthritis.

## Background

Cervical radiculopathy stems from degenerative disease in the cervical spine and seriously affects the quality of life of patients with upper limb pain or motor dysfunctions [1, 2]. It is characterized by circumferential narrowing of the cervical foramen and subsequent neural compression [3]. During the process of cervical radiculopathy, inflammation mediators were released [4, 5]. Neurogenic inflammation caused by mechanical compression of the cervical nerve root was reported to be the main cause of spastic pain [6–8]. Thus, anti-inflammatory analgesics are commonly used for the treatment of arthropathy, such as non-steroidal anti-inflammatory drugs and steroids [9, 10]. In addition, the inflammation mediators released can also cause the development of other joint diseases, such as osteoarthritis (OA). Inflammatory molecules secreted by the inflammatory synovium are key mediators of the disruptive processes related to arthritis pathophysiology. Interleukin (IL) and tumor necrosis factor (TNF) control the degeneration of articular cartilage matrix, and, therefore, are the main targets of therapeutic interventions [11]. TNF-α and IL-6 appear to be the main pro-inflammatory cytokines involved in the pathophysiology of OA [12, 13]. The current treatments for joint diseases mainly focus on the pain control and joint function improvement.

**Fig. 1:**
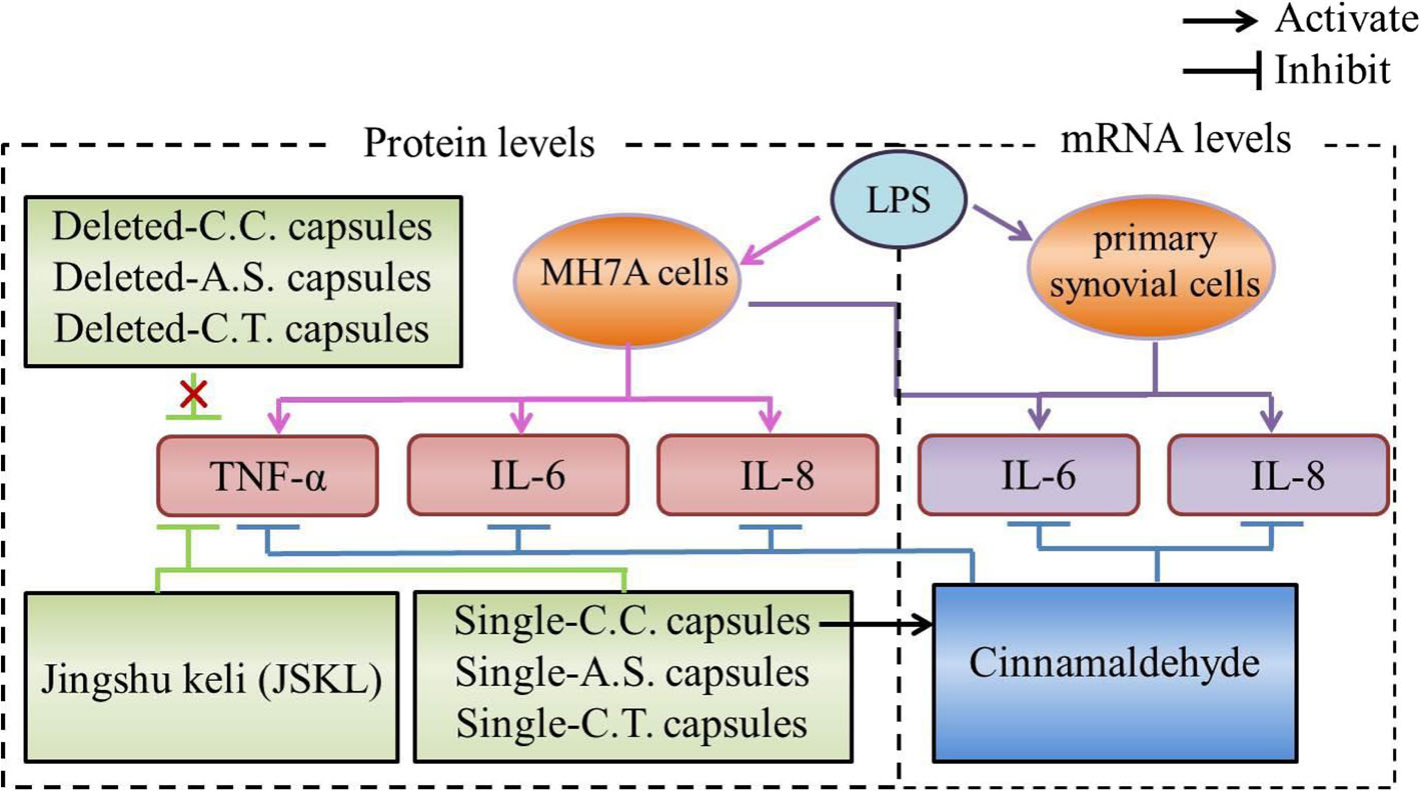
Schematic illustration of evaluation of the anti-inflammatory effects of JSKL and its components *in vitro*

MH7A cells, a human rheumatoid arthritis synovial cell line, exhibit characteristics of inflammatory cells and are often used for establishing RA cell models induced by lipopolysaccharide (LPS) [14, 15]. LPS is a component of the cell wall of Gram-negative bacteria [16]. LPS can stimulate MH7A cells to produce pro-inflammatory cytokines, such as TNF-α, interleukin 6 (IL-6) and interleukin 8 (IL-8) [17–19]. The Schematic of technology roadmap for assessing the anti-inflammatory effects of JSKL and its compounds *in vitro* is illustrated in Fig. 1.

*Jingshu Keli* (JSKL) is one of the commonly used drugs for the treatment of cervical spondylotic radiculopathy in China. It consists of seven traditional Chinese medicines, *Panax Notoginseng* (Burkill) F. H. Chen ex C. H. (*P.N.*), *Angelica Sinensis* (Oliv.) Diels (*A.S.*), *Ligusticum Chuanxiong* Hort. (*L.C.*), *Carthamus Tinctorius* L. (*C.T.), Gastrodia Elata* Bl. (*G.E.*), *Cinnamomum Cassia* Presl (*C.C.*) and *Bovis Calculus* Artifactus (*B.C.*) [20]. Some clinical validity data have been accumulating [21], and our previous preclinical studies found that JSKL attenuated C7 spinal nerve ligation-induced mechanical allodynia in rats by suppressing the activation of spinal microglia and p-Stat3 [22]. The aim of this study was to confirm the anti-inflammatory effect of JSKL and identify the contributing herbal components and compounds in it by examining the effects of JSKL, single-herb capsules, single-herb-deleted JSKL capsules and reference compounds for quality control in JSKL, as indicated by 2015 Chinese pharmacopoeia, on the expressions of TNF-α, IL-6 and IL-8 in the culture medium of LPS-induced MH7A cells.

## Materials and methods

### Sample preparation

JSKL, single-herb-deleted capsules and single-herb capsules were prepared by using the following methods. Into a 2.5 L four-necked flask equipped with an overhead bladed stirrer, distiller, and thermometer, 66.6 g *A.S.*, 66.6 g *L.C.*, 44.4 g *C.C.* and 1.5 L distilled water were added. The mixture was heated to the boiling point of water and then heated for 4 h to obtain volatile oil. The volatile oil was encapsulated with 4.2 g β-cyclodextrin. The remaining mixture in the flask was filtered using a 120-mesh filter to obtain Chinese herbal water 1 and dregs 1 and stored separately. 66.6 g *P.N.*, 66.6 g *G.E.* and 1.0 L distilled water were added into the same flask. The mixture was heated for 1 h and then filtered using a 120-mesh filter to obtain a mixture of Chinese herbal water 2 and dreg 2 and stored separately. The dregs 1, dregs 2 and *C.T.* were mixed and heated 2 times, with the first time, water was added 3 times and the sample was cooked for 1 h, and with the second time, water was added 3 times and the sample was cooked for 0.5 h. After cooled to room temperature, the mixture was filtered through a 120-mesh filter to get Chinese herbal water 3. The Chinese herbal water 1, 2 and 3 were mingled and the resultant sample was concentrated to a relative density of 1.30 (at 50 to 60 °C) using a rotary evaporator. Finally, *Bovis Calculus* Artifactus (18.5 g), volatile oil and dextrin were added to make a final sample. After drying and pulverizing, a compound sample of JSKL (about 200 g) was obtained. With the aforementioned method, a Chinese herbal medicine was removed in the preparation process to obtain single-herb-deleted capsules. With single-herb capsules, only one Chinese herbal medicine was added. The above-mentioned TCM granules were prepared by Sinopharm Group Jingfang Pharmaceutical Co., Ltd., Anhui, China. 

### Cell culture

The human synovial cell line MH7A was purchased from Riken Cell Bank (Ibaraki, Japan) and cultured in RPMI-1640 medium (Hyclone, USA) with 10% fetal bovine serum (FBS, Thermo, USA) and 1% penicillin/streptomycin (P/S, Hyclone, USA) at 37 °C in 5% CO_2_ humidified atmosphere [23]. Primary synovial cells from a rheumatoid arthritis patient were kindly provided by Dr. Qingwen Wang from Peking University Shenzhen Hospital, Shenzhen, China. The cells were incubated in DMEM medium (Hyclone, USA) with 10% FBS (Gibco, GrandIsland, USA), and P/S (1:100, Hyclone, USA) at 37 °C and in 5% CO_2_ [24]_._

### Sample dilution

One milligram sample was dissolved in 0.5 mL water. After mixing, 0.5 mL dimethyl sulfoxide (DMSO) was added. Upon centrifugation, supernatant was taken as stock solution. The monomer compounds were dissolved in DMSO to obtain 10 mmol/L stock solutions.

### CCK-8 assay

MH7A cells (4000 cells/well) were seeded into 96-well plates for 12 h and then treated with various doses of samples (JSKL, single-herb-deleted capsules, single-herb capsules and monomer compounds). The cells were incubated in 1640 medium with 10% FBS and 1% P/S for another 48 h. Then, Cell Counting Kit-8 (CCK-8, Dojindo Molecular Technologies, Kyushu, Japan) was added into each well and the plates were incubated at 37 °C for 2 h, according to the manufacturer’s operating instructions. The plates were placed onto an ELISA reader (Bio-Rad, LabWrench, Canada) and the optical density (OD) value of each well was analyzed at 450 nm.

### ELISA analysis

MH7A cells (5 × 10^5^ cells/well) were seeded into 6-well plates and cultured for 24 h. Then, the cells were treated by various doses of samples for 2 h and induced by 10 ng/mL LPS for 24 h. The cell culture supernatant was collected. The concentrations of TNF-α, IL-6 and IL-8 in the culture supernatant were determined by using the corresponding Human ELISA Kits. The OD value was measured at 450 nm. Then, the relative expressions (RE) of TNF-α, IL-6 and IL-8 were calculated, respectively, according to the following formula:

$$ RE\ \left(\%\right)= OD(treatment)/ OD(control)\times 100 $$

where, OD (treatment) is the OD value of the various doses of samples treatment group; and OD (control) is the OD value of the DMSO control group.

### Real-time polymerase chain reaction (real-time PCR)

MH7A cells or primary synovial cells were pretreated with of CIN for 2 h, and then incubated for another 6 h with 10 ng/mL LPS. Total RNAs were isolated using the commercial total RNA miniprep kit (Axygen, USA), according to the manufacturer’s instructions. Each sample was reversely transcribed using the cDNA synthesis kit (TaKaRa, China), by following the manufacturer’s protocol. The primer sequences were used for real-time PCR as shown in Table 1. Real-time PCR was performed using SYBR Green PCR Premix Ex Taq II reagents (TaKaRa) on a Light Cycler 480 II real-time system (Roche, USA), with GAPDH serving as house-keeping gene for normalization [25].

**Table 1:** Primer sequences for real-time PCR

Gene	Forward primer (5′ → 3′)	Reverse primer (5′ → 3′)
IL-6	CCTGACCCAACCACAAATGC	ATCTGAGGTGCCCATGCTAC
IL-8	GGTGCAGTTTTGCCAAGGAG	TTCCTTGGGGTCCAGACAGA
TNF-α	CCCCAGGGACCTCTCTCTAATC	GGTTTGCTACAACATGGGCTACA
GAPDH	GGAGTCCACTGGCGTCTT	AGGCTGTTGTCATACTTCTCAT

### Statistical analysis

All data were expressed as means ± standard derivations (SD) from three independent experiments. Statistical significance between groups was analyzed by employing ANOVA of the GraphPad Prism 6.0 (GraphPad Software Inc., San Diego, CA, USA). Statistical significance was set at *p* < 0.05.

## Results

### Safe concentrations of JSKL samples on MH7A cells

In order to determine the safe concentrations of JSKL, single-herb capsules, single-herb-deleted capsules on MH7A cells, CCK-8 assay was used to detect the cytotoxicity of these samples at different concentrations. As shown in Table 2, JSKL with concentrations of 0.02 and 0.2 μg/mL could promote the proliferation of MH7A cells, compared with the control group. JSKL and single-herb-deleted capsules showed no cytotoxicity at given concentrations. So, the maximum safe concentration of JSKL and single-herb-deleted capsules was 2 μg/mL. When the concentration of *C.C.* was 2 μg/mL, the relative growth rate of MH7A cells was only 30 ± 3.5%, which inhibited the proliferation of MH7A cells. When the concentration of *C.C.* was 0.2 μg/mL, the relative growth rate of MH7A cells was 95.98 ± 4.3%. So, the maximum safe concentration of *C.C.* was 0.2 μg/mL. Other herbs (*C.T.*, *A.S.*, *L.C.*, *G.E.*, *P.N.*) showed no cytotoxicity at any given concentrations. So, their concentrations were set at 2 μg/mL in the following studies. As shown in Table 3, the safe concentrations of the monomeric compounds ferulic acid and CIN were 0.1 μM and 0.5 μM, respectively. The safe concentration of gastrodin, cholic acid, notoginsenoside R1, ginsenoside Rg1 and ginsenoside Rb1 was 10 μM.

**Table 2:** Effects of different drug concentrations of JSKL, deleted-herb-capsule and single-herb-capsule on MH7A cells activity

Samples	Drug concentrations (μg/mL)			
	0	0.02	0.2	2
JSKL	1.430 ± 0.073	1.692 ± 0.197*	1.633 ± 0.165*	1.538 ± 0.192
Deleted *C.C.* capsules	0.893 ± 0.057	0.911 ± 0.078	0.861 ± 0.102	1.020 ± 0.125
Deleted *C.T.* capsules	1.017 ± 0.104	1.025 ± 0.142	1.031 ± 0.092	1.224 ± 0.112*
Deleted *A.S.* capsules	1.017 ± 0.104	1.065 ± 0.107	1.001 ± 0.046	1.028 ± 0.195
Deleted *L.C.* capsules	1.017 ± 0.104	1.134 ± 0.088	1.180 ± 0.048*	1.099 ± 0.097
Deleted *G.E.* capsules	0.893 ± 0.065	1.063 ± 0.084*	1.029 ± 0.095	1.049 ± 0.092*
Deleted *P.N.* capsules	1.430 ± 0.073	1.393 ± 0.075	1.435 ± 0.047	1.391 ± 0.040
single-*C.C.*	1.413 ± 0.056	1.390 ± 0.157	1.353 ± 0.106	0.429 ± 0.121**
single-*C.T.*	1.385 ± 0.098	1.344 ± 0.080	1.489 ± 0.142	1.580 ± 0.141*
single-*A.S.*	1.385 ± 0.098	1.402 ± 0.096	1.385 ± 0.148	1.426 ± 0.158
single-*L.C.*	1.385 ± 0.098	1.337 ± 0.034	1.282 ± 0.023	1.464 ± 0.090
single-*G.E.*	1.413 ± 0.056	1.417 ± 0.066	1.421 ± 0.041	1.294 ± 0.140
single-*P.N.*	1.430 ± 0.073	1.450 ± 0.153	1.383 ± 0.139	1.600 ± 0.063*

Note: 0 μM was defined as the control group. Data are expressed as means ± SD (*N* = 5). *p < 0.05, **p < 0.01 vs control group

**Table 3:** Effects of monomeric compounds with different JSKL concentrations on the activity of MH7A cells

Samples	JSKL Concentrations (μM)			
	0	0.1	1	10
Ferulic acid	1.930 ± 0.044	2.048 ± 0.025	0.817 ± 0.111**	0.255 ± 0.072**
CIN_(1/20)_	1.430 ± 0.073	1.692 ± 0.197*	1.633 ± 0.165*	1.538 ± 0.192
Cholic acid	1.140 ± 0.051	1.090 ± 0.122	0.997 ± 0.053*	1.118 ± 0.074
Gastrodin	1.930 ± 0.099	1.973 ± 0.032	1.934 ± 0.021	1.987 ± 0.089
Notoginsenoside R1	1.723 ± 0.039	2.018 ± 0.413	2.281 ± 0.379*	2.046 ± 0.094*
Ginsenoside Rg1	1.723 ± 0.17	2.007 ± 0.052*	2.257 ± 0.152*	1.685 ± 0.147
Ginsenoside Rb1	1.723 ± 0.017	1.942 ± 0.110*	2.184 ± 0.151*	2.074 ± 0.131*

Note:1/20: the actual concentration of CIN was 1/20 times of the label concentration, which means 0, 0.005, 0.05, 0.5 μM from left to right. 0 μM was defined as the control group. Data are expressed as means ± SD (N = 5). *p < 0.05, **p < 0.01 *vs*. control group

### Effect of JSKL on the expression of pro-inflammatory cytokines in MH7A cells stimulated by LPS

**Fig. 2:**
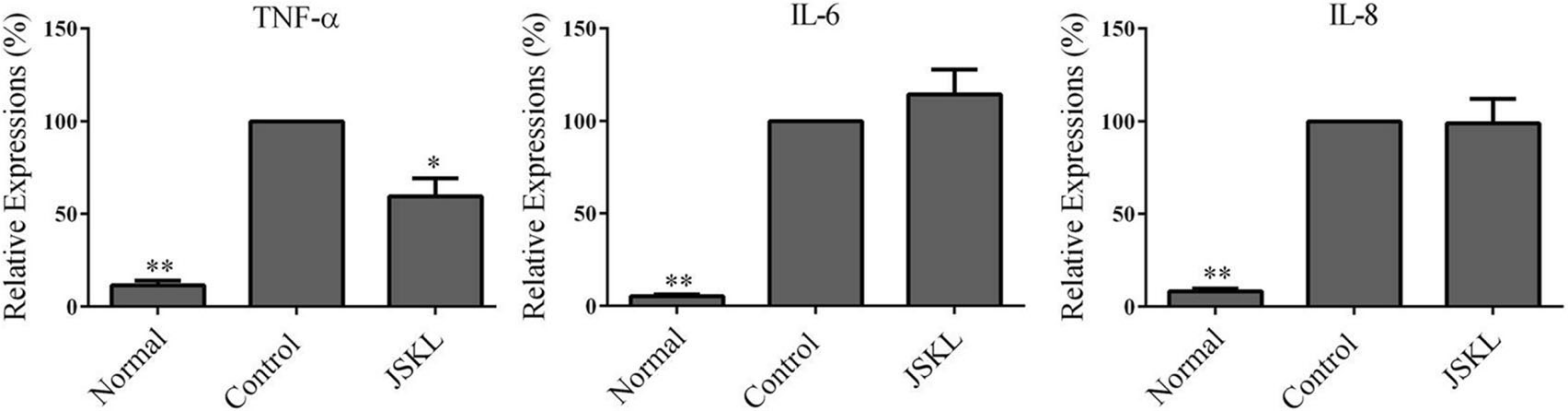
Effect of JSKL on the expression of pro-inflammatory cytokines in MH7A cells stimulated by LPS. *p < 0.05, **p < 0.01 vs control group

As shown in Fig. 2, the expression of TNF-α, IL-6 and IL-8 was significantly increased after MH7A cells were stimulated by LPS (*p* < 0.05). JSKL (2 μg/mL) significantly inhibited the expression of TNF-α (*p* < 0.05) but exerted no significant effect on the expressions of IL-6 and IL-8 (*p* > 0.05).

### Effect of JSKL, single-herb and single-herb-deleted capsules on the expression of TNF-α, IL-6 and IL-8 in MH7A cells stimulated by LPS

**Fig. 3:**
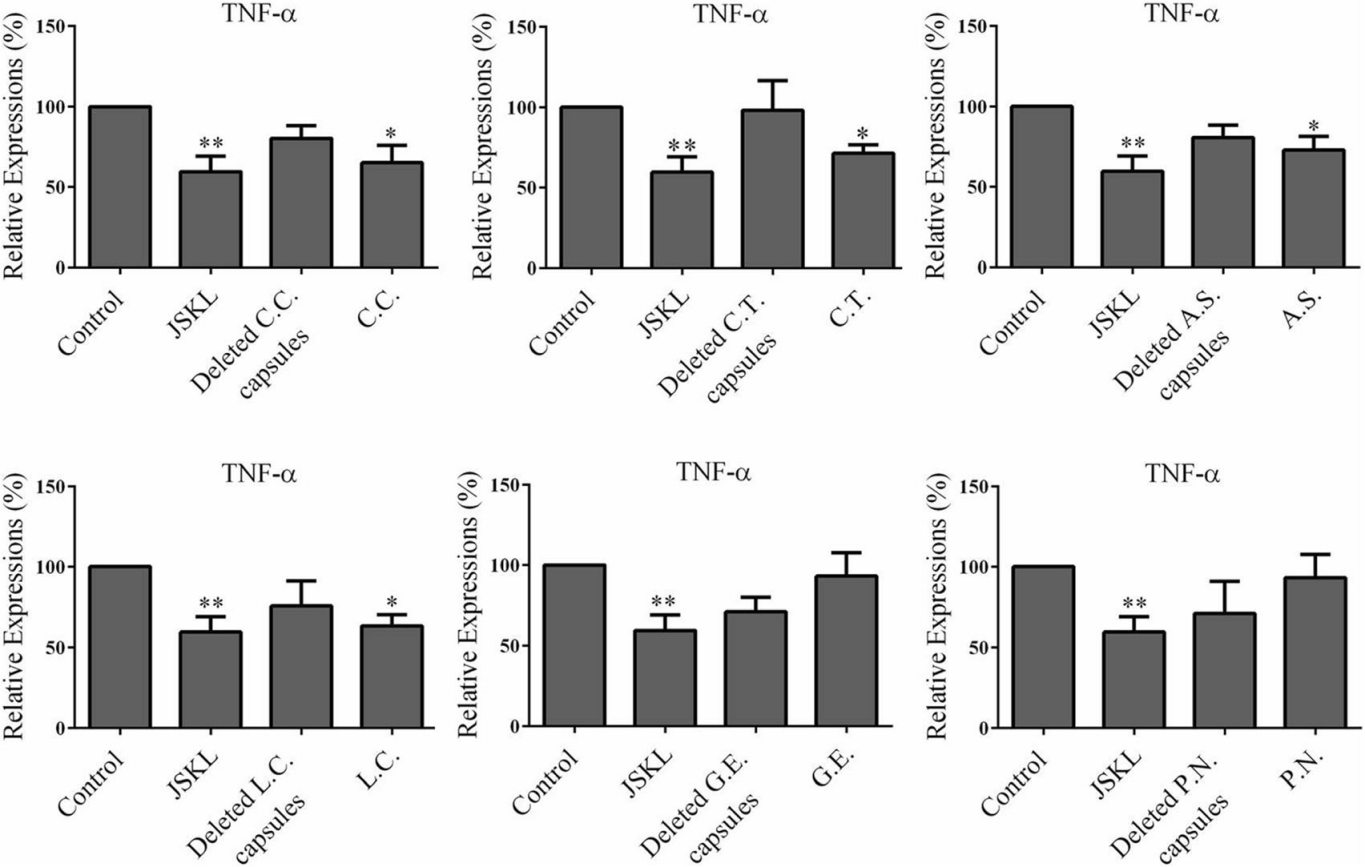
Effect of JSKL, deleted-herb-capsule and single-herb-capsule on the expression of TNF-α in LPS-stimulated MH7A cells. *p < 0.05, **p < 0.01 vs control group

**Fig. 4:**
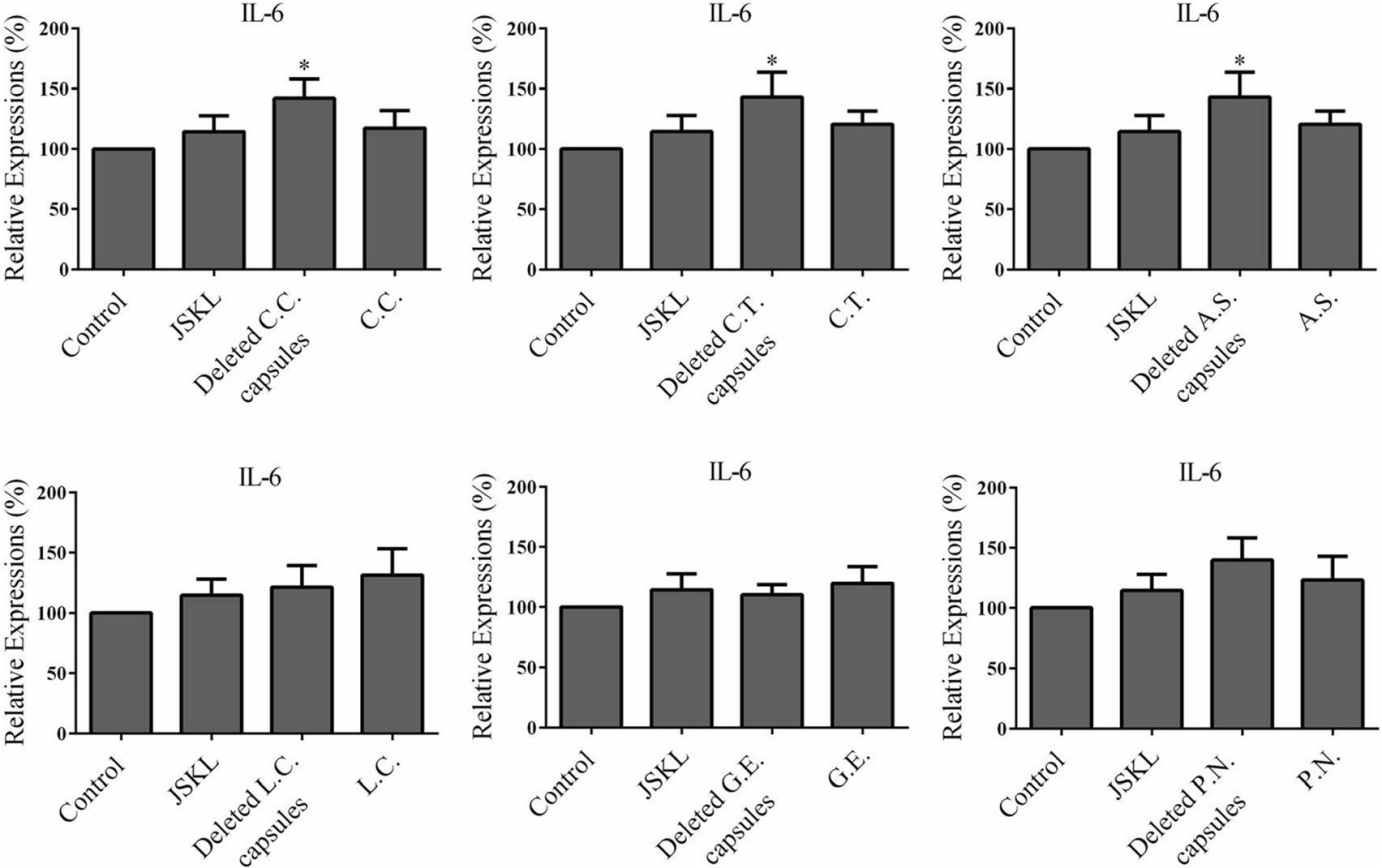
Effect of JSKL, deleted-herb-capsule and single-herb-capsule on the expression of IL-6 in LPS-stimulated MH7A cells. *p < 0.05 vs control group

**Fig. 5:**
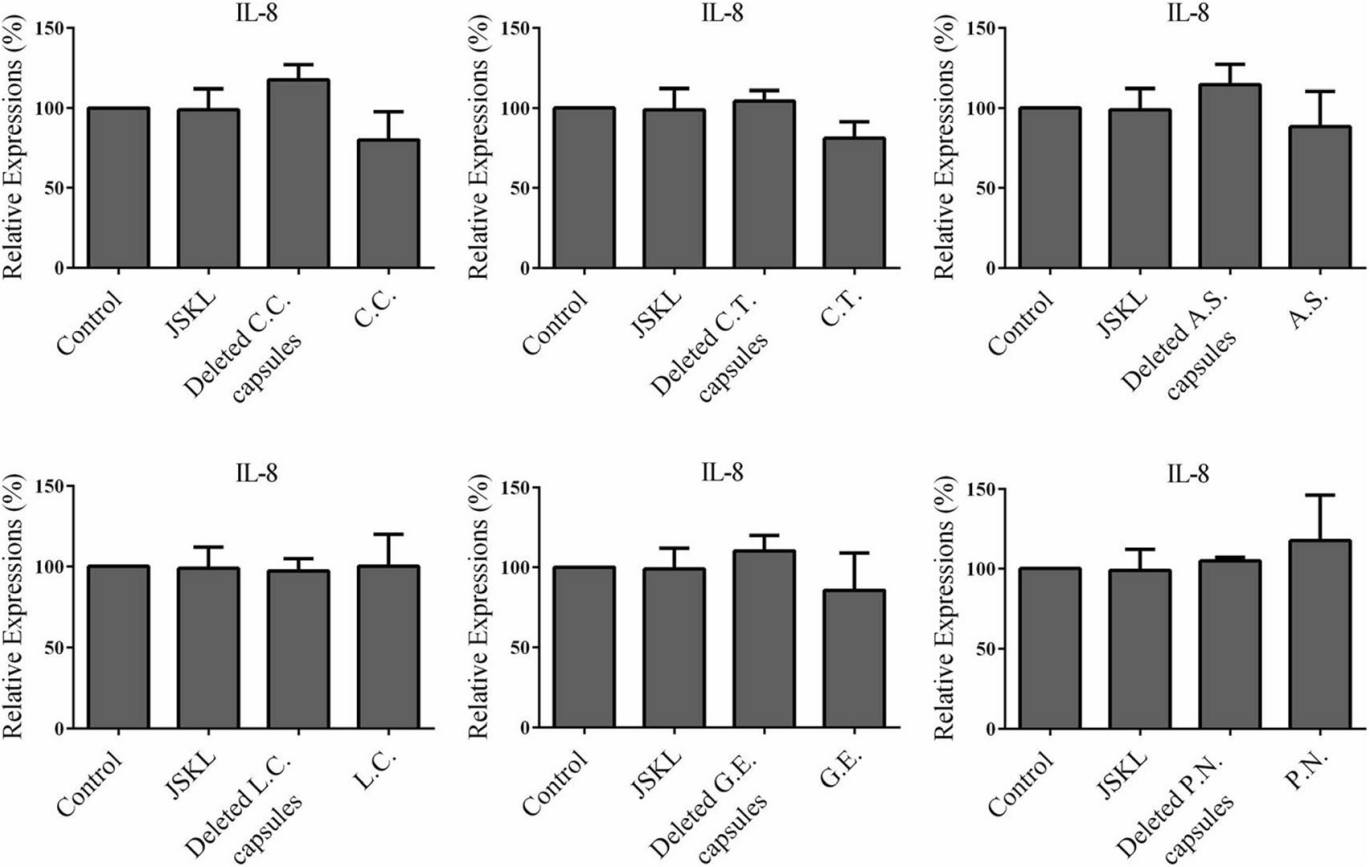
Effect of JSKL, deleted-herb-capsule and single-herb-capsule on the expression of IL-8 in LPS-stimulated MH7A cells

As shown in Fig. 3, compared with control group, JSKL, single-*C.C.*, single-*C.T.*, single-*A.S.*, single-*L.C.* could significantly inhibit the expression of TNF-α in MH7A cells (*p* < 0.05). In contrast, single-herb-deleted capsules did not inhibit the expression of the TNF-α in MH7A cells (*p* > 0.05). So, the *C.C.*, *C.T.* and *A.S.* components in JSKL were essential for the inhibitory effect on the expression of TNF-α in MH7A cells. As shown in Fig. 4, compared with the control group, JSKL and single-herb capsules had no significant effect on the expression of pro-inflammatory cytokine IL-6 (*p* > 0.05). However, single-*C.C.*-deleted capsules, single-*C.T.*-deleted capsules and single-*A.S*.-deleted capsules could promote the expression of pro-inflammatory cytokine IL-6 in MH7A cells (*p* < 0.05). So, *C.C.*, *C.T.* and *A.S.* in JSKL didn’t promote the expression of IL-6 in MH7A cells. As shown in Fig. 5, the samples had no significant effect on the expression of IL-8 in MH7A cells, compared with the control group (*p* > 0.05).

### Effects of single pure compounds on the expression of TNF-α, IL-6 and IL-8 in MH7A cells

**Fig. 6:**
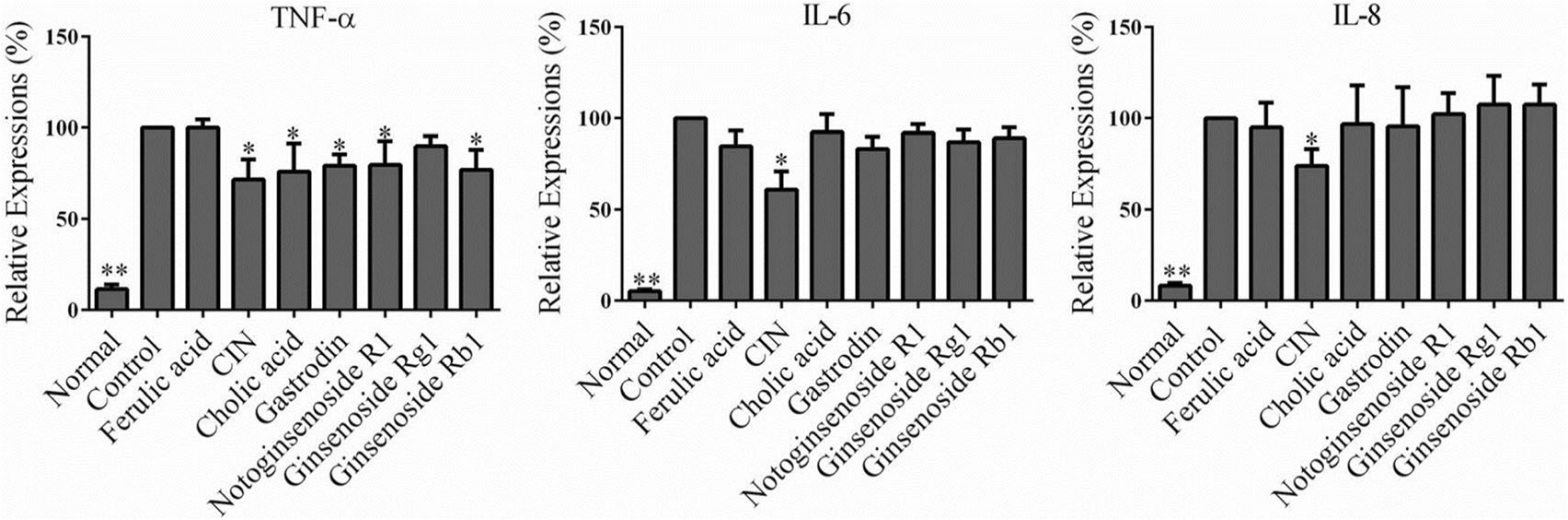
Effect of single pure compounds on the expression of TNF-α, IL-6 and IL-8 in LPS-stimulated MH7A cells. *p < 0.05, **p < 0.01 *vs*. control group

Cinnamaldehyde (CIN), ferulic acid, gastrodin, cholic acid and notoginsenoside R1, ginsenoside Rg1, ginsenoside Rg1, are the indicated reference compounds for quality control of JSKL stipulated in 2015 Chinese pharmacopoeia. As shown in Fig. 6, CIN inhibited the expression of TNF-α, IL-6 and IL-8 in MH7A cells stimulated by LPS (*p* < 0.05). Cholic acid, gastrodin, notoginsenoside R1 and ginsenoside Rb1 significantly reduced the expression of TNF-α (*p* < 0.05) without affecting the expression of IL-6 and IL-8 (*p* > 0.05). So, CIN in *C.C.* had the most potent anti-inflammatory effect among these reference compounds.

### Effects of CIN on the suppressive releases of IL-6 and IL-8 in LPS-stimulated MH7A cells and LPS-stimulated primary synovial cells

**Fig. 7:**
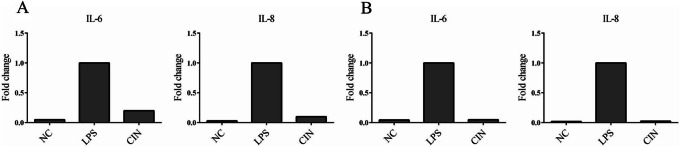
Anti-inflammatory effects of CIN in LPS-stimulated MH7A cells and primary synovial cells. Realtime PCR results of 0.5 μM of CIN on the gene expression of IL-6 and IL-8 in LPS-stimulated MH7A cells (**a**) and LPS-stimulated primary synovial cells (**b**)

MH7A cells were pretreated with 0.5 μM CIN for 2 h, and then stimulated with TNF-α (20 ng/mL). After 6 h, the mRNA levels of IL-6 and IL-8 were measured by qPCR. As shown in Fig. 7a, LPS significantly upregulated IL-6 and IL-8 gene expressions, while 0.5 μM CIN dramatically inhibited the gene expression of both IL-6 and IL-8.

We also investigated the anti-inflammatory effects of CIN in primary synovial cells. The results (Fig. 7b) showed that LPS obviously stimulated the secretions of IL-6 and IL-8 in primary synovial cells.

## Discussion

JSKL is one of the TCM herbal medicines for the treatment of cervical spondylotic radiculopathy. In previous studies, we established a rat model of cervical spondylotic radiculopathy by C7 spinal nerve ligation (SNL) with 6–0 silk suture. We found that JSKL could inhibit SNL-induced allodynia and microglia activation [22]. In this study, MH7A cells were used to confirm that JSKL was able to inhibit expression of the main pro-inflammatory factor TNF-α, and three herbal components (*C.C.*, *C.T.* and *A.S.)* played essential roles in the action. Further studies demonstrated that CIN from *C.C.* was the most potent single compound responsible for suppressing LPS-induced and TNF-α-induced expressions of IL-6 and IL-8 in MH7A cells.

TNF-α is a pro-inflammatory cytokine involved in cellular activation and inflammation, and is mainly found in neuromembrane cells and vascular endothelial cells [26–28]. Because neuromembrane cells are closely connected with nerve fibers, it is believed that TNF-α may be related to neurogenic pain [29]. The current study, for the first time, clarified that JSKL could inhibit the expression of TNF-α in LPS-induced MH7A cells.

This study examined the effects of JSKL, single-herb and single-herb-deleted capsules on the expression of pro-inflammatory cytokines in MH7A cells. It was found that JSKL could inhibit the expression of TNF-α, while those without *C.C.*, *C.T.*, *A.S.* and *L.C.* could not reduce the expression of TNF-α. At the same time, single herbal component, i.e., *C.C.*, *C.T.*, *A.S.* and *L.C.* could also inhibit the expression of TNF-α, indicating that *C.C.*, *C.T.* and *A.S.* are the main components of JSKL that inhibit the expression of TNF-α. Similarly, JSKL did not affect the expression of IL-6, while JSKL containing no *C.C.*, *C.T.* and *A.S.*, could increase the expression of IL-6, suggesting that *C.C.*, *C.T.* and *A.S.* are important medicinal components or ingredients. On the basis of the findings, we conclude that *C.C.*, *C.T.* and *A.S.* are the main anti-inflammatory herbs of JSKL.

In this study, we found that *C.T.* inhibited the expression of TNF-α and IL-8 in MH7A cells. It has been reported that saffower yellower, the main component of *C.T.*, has anti-inflammatory effects [30]. Saffower yellower was also shown to reduce the levels of TNF-α, IL-6, IL-1β and COX-2, inhibit the expression of NF-κΒ P65 protein and NF-κΒ P65 gene, and possess anti-inflammatory effect in rats with radicular lumbar spondylosis [31]. *A.S.* can also inhibit the expression of TNF-α in MH7A cells. Pang *et al*. [32] established rat inflammation model by intraperitoneal injection of LPS and found that water-soluble low molecular substances in *A.S.* could significantly reduce the levels of TNF-α and IL-6 in plasma. Ferulic acid, an indicator component of *A.S.*, showed no anti-inflammatory activity at the determined concentration, suggesting that other active compounds might play an anti-inflammatory role in *A.S.*.

CIN, the main component of *C.C.*, significantly decreased the expression of pro-inflammatory cytokines TNF-α, IL-6 and IL-8. Zhang *et al*. [33] found that CIN could significantly inhibit the expression of prostaglandin E2 and nitric oxide, down-regulate the expression of membrane-related prostaglandin synthase-1 and cyclooxygenase in RAW264.7 cells stimulated by LPS, and had obvious anti-inflammatory and antipyretic effects. The findings suggest that *C.C.* is an important anti-inflammatory TCM herbal component that can be included in the formulation of JSKL capsule, and cinnamaldehyde, the monomer component, was identified to be the key active ingredient for its anti-inflammatory action.

In this paper, CCK-8 assay showed that the safe concentration of JSKL was up to 2 μg/mL with MH7A cells. One limitation of the study is that no data were provided on its *in vivo* safety. In a previous study, we found that JSKL at the dose of 400 mg/day/rat attenuated C7 spinal nerve ligation (SNL)-induced mechanical allodynia in rats, without obvious side-effects after 14-day treatment [22]. Furthermore, the bioactive compound CIN at the dose of 75 mg/kg/day alleviated collagen-induced arthritis in rats, without conspicuous side-effects after a 21-day treatment [34]. Future studies with longer treatment time and organ-specific analysis are warranted to evaluate the long-term safety of JSKL.

## Conclusions

This study demonstrated that JSKL had anti-inflammatory effect *in vitro; C.C.*, *A.S.* and *C.T.* were the main and essential herbal components in JSKL responsible for this action; CIN from *C.C.* was one the most potent single compounds among indicator components specified in 2015 Chinese pharmacopoeia. This *in vitro* study provided solid evidence that JSKL can be clinically used for prevention and treatment of inflammatory disorders. Furthermore, CIN has potential to be used for the treatment of some inflammation-related orthopedic diseases, such as rheumatic arthritis and osteoarthritis.

## Data Availability

Data sharing is not applicable to this article as no datasets were generated or analysed during the current study.

## Abbreviations

*A.S*.: *Angelica Sinensis (Oliv.) Diels*
*B.C.*: *Bovis Calculus Artifactus*
*C.C.*: *Cinnamomum cassia Presl*
*C.T.*: *Carthamus tinctorius L.*
*G.E.*: *Gastrodia Elata Bl.*
IL-6: Interleukin 6
IL-8: Interleukin 8
JSKL: Jingshu Keli
*L.C.*: *Ligusticum Chuanxiong Hort.*
OA: Osteoarthritis
*P.N.*: *Panax Notoginseng (Burkill) F. H. Chen ex C. H.*
TCM: Traditional Chinese medicine
TNF-α: Tumor necrosis factor-alpha
